# Bio‐Inspired Polyanionic Electrolytes for Highly Stable Zinc‐Ion Batteries

**DOI:** 10.1002/anie.202311268

**Published:** 2023-09-01

**Authors:** Haobo Dong, Xueying Hu, Ruirui Liu, Mengzheng Ouyang, Hongzhen He, Tianlei Wang, Xuan Gao, Yuhang Dai, Wei Zhang, Yiyang Liu, Yongquan Zhou, Dan J. L. Brett, Ivan P. Parkin, Paul R. Shearing, Guanjie He

**Affiliations:** ^1^ Electrochemical Innovation Lab Department of Chemical Engineering University College London Torrington Place London WC1E 7JE UK; ^2^ Christopher Ingold Laboratory Department of Chemistry University College London 20 Gordon Street London WC1H 0AJ UK; ^3^ Key Laboratory of Comprehensive and Highly Efficient Util Laboratory of Salt Lake Resources Chemistry of Qinghai Province Chinese Academy of Sciences Xining Qinghai 810008 China; ^4^ Department of Earth Science and Engineering Imperial College London SW7 2AZ UK

**Keywords:** Bio-Inspired Electrolyte, Interface Engineering, Zinc-Ion Batteries, in situ SEI

## Abstract

For zinc‐ion batteries (ZIBs), the non‐uniform Zn plating/stripping results in a high polarization and low Coulombic efficiency (CE), hindering the large‐scale application of ZIBs. Here, inspired by biomass seaweed plants, an anionic polyelectrolyte alginate acid (SA) was used to initiate the in situ formation of the high‐performance solid electrolyte interphase (SEI) layer on the Zn anode. Attribute to the anionic groups of −COO^−^, the affinity of Zn^2+^ ions to alginate acid induces a well‐aligned accelerating channel for uniform plating. This SEI regulates the desolvation structure of Zn^2+^ and facilitates the formation of compact Zn (002) crystal planes. Even under high depth of discharge conditions (DOD), the SA‐coated Zn anode still maintains a stable Zn stripping/plating behavior with a low potential difference (0.114 V). According to the classical nucleation theory, the nucleation energy for SA‐coated Zn is 97 % less than that of bare Zn, resulting in a faster nucleation rate. The Zn||Cu cell assembled with the SA‐coated electrode exhibits an outstanding average CE of 99.8 % over 1,400 cycles. The design is successfully demonstrated in pouch cells, where the SA‐coated Zn exhibits capacity retention of 96.9 % compared to 59.1 % for bare Zn anode, even under the high cathode mass loading (>10 mg/cm^2^).

## Graphical abstract

Inspired by biomass seaweed plants, an anionic polyelectrolyte alginate acid (SA) was used to initiate the in situ formation of the high‐performance solid electrolyte interphase (SEI) layer on the Zn anode. Attribute to the anionic groups of −COO^−^, the affinity of Zn^2+^ ions to alginate acid induces a well‐aligned accelerating channel for uniform plating. The SEI also regulates the desolvation structure of Zn^2+^ and facilitates the formation of compact Zn (002) lattice planes.

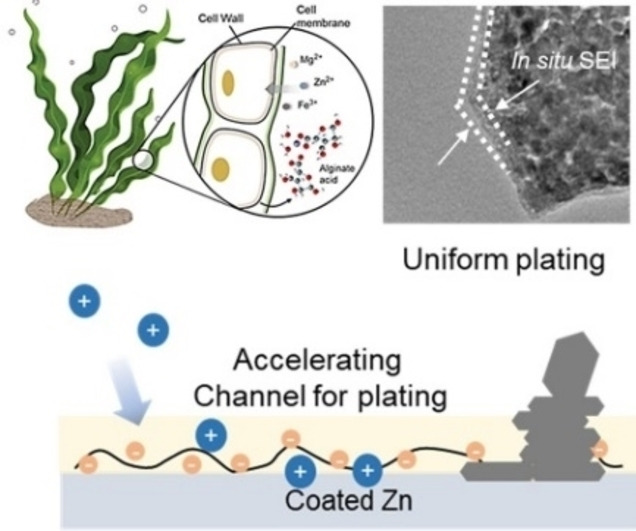

## Introduction

Aqueous multivalent‐ion batteries (Zn^2+^, Mg^2+^, Ca^2+^, Al^3+^) have been expected as next‐generation energy storage systems for large‐scale applications due to their natural abundance, low manufacturing cost, and high ionic conductivity.^[1]^ Among them, aqueous Zinc‐ion batteries (ZIBs) attract considerable attention because the Zn anode has advantages including high energy density (5855 mAh mL^−1^), suitable reduction potential (−0.762 V vs. standard hydrogen electrode (SHE)), and nontoxicity.^[2]^ However, severe interfacial side reactions on the Zn metal anode result in dendrite formation, hydrogen evolution reaction (HER) and corrosion, which reduce the reversibility and stability of the Zn anode and limit the real‐life application of ZIBs.^[3]^

To improve the overall electrochemical performance of the Zn anode, various approaches have been proposed to induce a stable solid electrolyte interphase (SEI) layer by electrolyte engineering^[4]^ and surface modification.^[5]^ Because of the low operating voltage window of aqueous electrolytes, it is more difficult to form SEI layer on Zn anode in zinc‐ion batteries compared to that in lithium‐ion batteries.[^28^, ^29^] Strategies such as electrolyte modulation utilizing water‐in‐salt electrolytes or deep eutectic electrolytes reduce the free water molecules and regulate interfacial binding energy to form the stable SEI layer.^[6]^ The high‐concentration electrolytes and deep eutectic solvents strategies are at the early stage to realize cost‐effective and economically sustainable ZIBs, thus low‐concentration, salt‐in‐water electrolytes are still the research focus.^[7]^ Considering the availability of materials, ease of fabrication and interface manipulation, polyanionic interface modification is the most promising method as shown in the radar diagram (Figure S1). It could be consistent with the manufacturing process of the extrusion coater and inhibit the dendrite growth and side reactions by ex situ and in situ modifications. In situ modification is a more ideal method,^[8]^ whereas due to the high redox potential of Zn/Zn^2+^, designing an efficient in situ SEI layer for ZIBs is still a crucial challenge. This Zn/Zn^2+^ redox potential means the hydrogen evolution reaction (HER) and Zn deposition would take precedence over the reductive decomposition of most anions and organic solvents, resulting in the dendrite growth, corrosion, and passivation of the Zn anode.[^6^, ^8^, ^9^] Hence, it is of utmost importance for ZIBs to find cost‐effective and in situ methods to construct stable and efficient SEI layers.

**Scheme 1:**
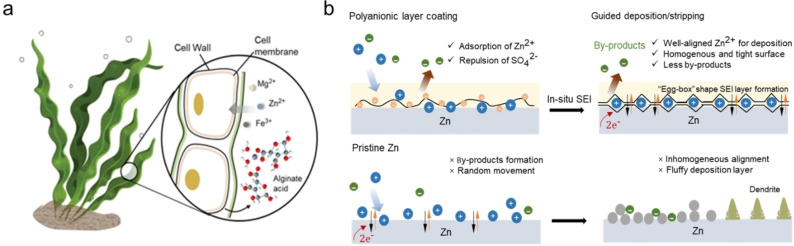
(a) Schematic diagram of bio‐inspired anionic polyelectrolyte from seaweed absorbing metal cations from the seawater. (b) Schematic diagrams of the bare Zn and the polyanionic electrolyte coated Zn plating behaviours, where polyanionic layer coating induces a well‐aligned Zn^2+^ accelerating channel, while bare Zn possesses a fluffy deposition.

**Figure 1:**
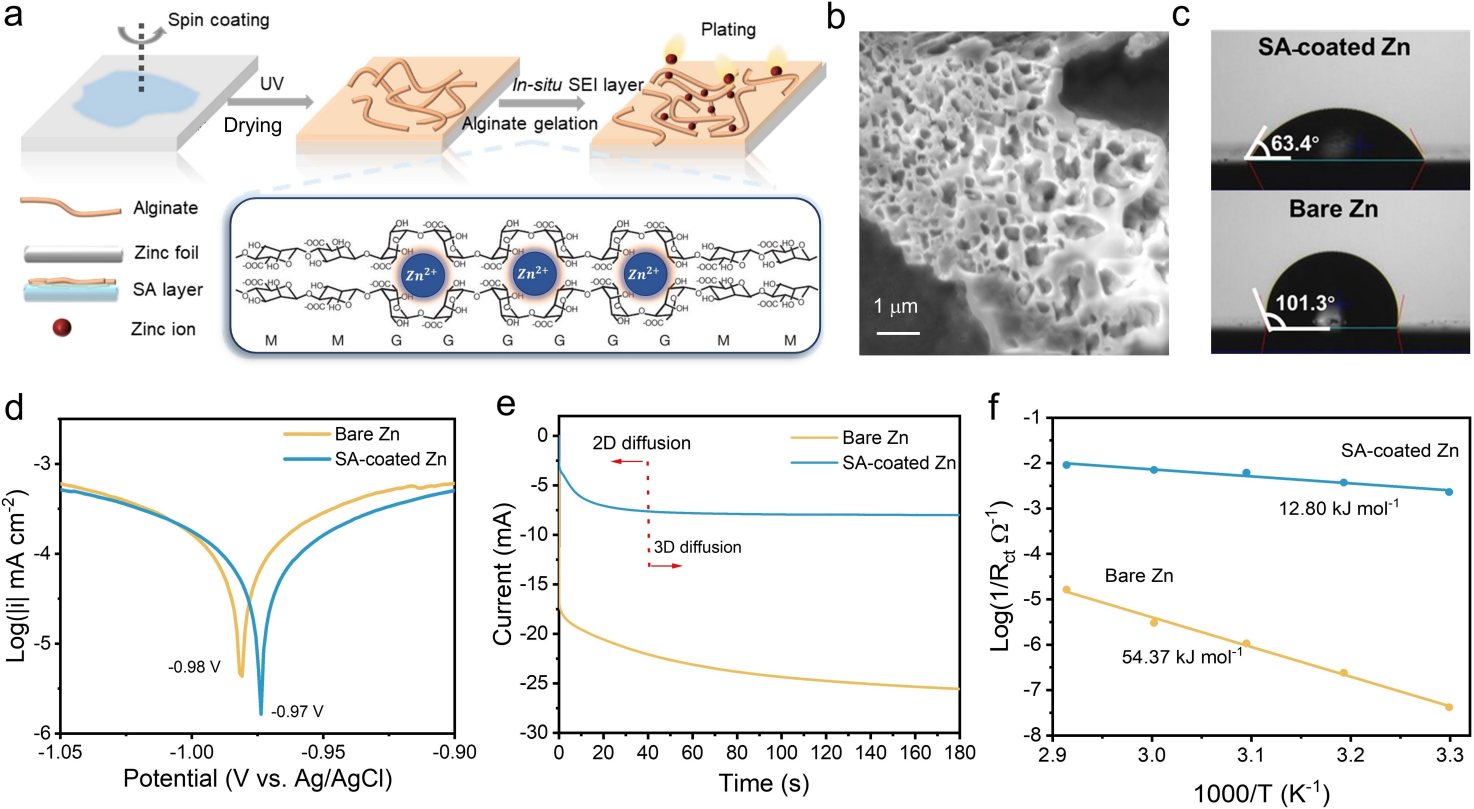
Preparation and characterization of the in situ formed anionic SEI layer. (a) Schematic diagram for the synthesis of the in situ SEI layer. (b) Scanning electron microscope (SEM) image of the anionic film showing the porous structure. (c) Contact angle measurements of 3 M ZnSO_4_ electrolyte on the surface of the coated and the bare Zn electrode. (d) Linear polarization curves of the SA‐coated Zn electrode and the bare Zn electrode. (e) Chronoamperometric (CA) curves of Zn plating on the SA‐coated Zn electrode and the bare Zn electrode at a −150 mV overpotential (f) The activation energy of both electrodes is calculated by the Arrhenius equation.

Seaweed, also known as kelp, is countless species of marine plants absorbing and accumulating various metal cations from the seawater, including essential metal cations such as Fe^3+^, Cu^2+^ and Zn^2+^ as shown in Scheme 1a. The negatively charged polysaccharides in the seaweed can bind with metal cations through process chelation to form stable complexes. Inspired by kelp‐cation accumulation and anti‐ageing properties, here, we propose a surface modification strategy to assist the formation of a robust in situ SEI by a bio‐inspired anionic layer. Sodium alginate (SA), a polysaccharide derived from kelp, contains free carboxyl and hydroxyl groups with D‐mannuronic (M) and L‐guluronic acid (G) as monomers. It can chelate with divalent cations (Zn^2+^, Ca^2+^, Ba^2+^, Al^3+^) by the block of G monomer forming the highly ionic and conductive “egg‐box” hydrogel structures.^[10]^ By coating SA on the Zn anode surface, an in situ SEI can be initiated during plating/stripping mechanism, where two SA single chains would in situ polymerize with Zn^2+^ by interlocking the Zn^2+^ ions in the electrolyte and construct a homogeneous Zn^2+^ diffusion layer (Figure 1a). This formation essentially establishes a hydrogel‐based protective layer over the Zn surface, enhancing its stability. The SA layer applied to the Zn anode functions as an anionic layer, governing the desolvation structure of Zn^2+^, which contributes to the preferred plating on the Zn (002) surface, resulting in the formation of a uniform and densely deposited layer. As shown in Scheme 1b, the SEI layer can construct an accelerating channel for uniform Zn plating in comparison to the pristine Zn anode. The well‐aligned Zn^2+^ cations facilitate a homogeneous and dense deposition, whereas there is a fluffy deposition for the bare Zn. Meanwhile, the in situ SEI featured an enhanced mechanical strength to encounter the dendrite formation along with the inorganic components, such as zinc hydroxide sulfate hydrate (ZHS). Correspondingly, it exhibits outstanding stability with an average Coulombic efficiency of up to 99.8 % over 1,400 cycles, and the in situ Raman profile has further confirmed its long‐term stability and reversibility. Implied with the commercial MnO_2_ cathode, the battery with the SA‐coated Zn anode exhibits an excellent specific capacity of 109.3 mAh g^−1^ with 87.1 % capacity retention over 3,000 cycles at 5 A g^−1^. Compared to other strategies, this work utilizing bio‐inspired polyanionic electrolyte can offer a merit voltage hysteresis, for which under the same current density, the biomass polyanionic coating exhibits the lowest overpotential. This anionic polyelectrolyte can be applied on both cathode and anode as a universal candidate for interface regulations.

## Results and Discussion

To construct a stable in situ SEI interface and enable a uniform Zn plating, the SA layer was prepared on the Zn foil by the spin coating method as illustrated in Supporting Information. The anionic COO^−^ in G monomers could absorb Zn^2+^ in the electrolyte and the in situ crosslink initiates zinc‐ion conductive alginate gelation. After the gelation process, the polyanionic layer inspired from the alginate eventually becomes an in situ SEI layer to protect the Zn anode (Figure 1a). The in situ formation process of the SEI layer was unveiled by Fourier Transform Infrared (FTIR) spectroscopy (Figure S2). Before cycling, the stretching vibration of the O−H bond for SA‐coated Zn electrode is about 3230 cm^−1^. The bands of the asymmetric and symmetric COO^−^ stretching vibration are around 1601 cm^−1^ and 1451 cm^−1^.^[11]^ After 15 cycles, the bands observed at 1079 cm^−1^ and 949 cm^−1^ become significant, which attribute to the C−O stretching vibration from the pyranosyl ring and the C−C−H/C−O‐H deformation, respectively.^[12]^ The O−H band after cycling become narrower than the initial state, from 3230 cm^−1^ to 3285 cm^−1^. Besides, the asymmetric stretching vibration of COO^−^ after cycling shifts to 1594 cm^−1^, while the symmetric stretching vibration of COO^−^ after cycling shifts to 1406 cm^−1^. This difference in a band stretching in COO^−^ and the O−H bond is due to the attraction of the Zn^2+^ ions in the egg‐box helical structure. The abundant O−H bonds and COO^−^ in the SA layer also could not only offer excellent hydrophilicity but could also coordinate the Zn^2+^ solvent sheath.^[13]^ As shown in Figure 1b, the attained coating exhibits a porous structure. Moreover, the contact angle of the SA‐coated Zn electrode in 3 M ZnSO_4_ is 63.4°, whereas that of bare Zn is 101.3° (see Figure 1c). This could be attributed to the hypothesis that hydrophilic SA‐coated Zn electrode would reduce the activation energy for the metal nucleation at the interface and hence regulate the uniform Zn plating/stripping.^[14]^

To unveil the interfacial mechanism of the bio‐inspired layer, the thermodynamic stability and ion‐transport kinetics were investigated. As shown in Figure 1d, the corrosion resistance performance was tested by the linear sweep voltammetry (LSV). Compared with the bare Zn electrode, the corrosion current density of the SA‐coated electrode is reduced by 19.39 %, and the corrosion potential is increased from −0.982 V to −0.973 V. Most notably, the corrosion current has also been reduced to −5.78 (log(mA/cm^2^)) compared to −5.33 (log(mA/cm^2^)) for the untreated Zn anode, which indicates the anionic layer could effectively reduce the corrosion rate and suppress the corrosion process.^[15]^ As for metal corrosion, reduced corrosion potential and current could result in a less tendency of a metal to corrode and a lower corrosion rate for H_2_ evolution, respectively.^[16]^ In addition, the in situ formed SEI layer even modifies the plating mechanism and dendrite formation. A 2D diffusion was attained for SA‐coated Zn (see Figure 1e).

Regarding the diffusion kinetics, the current for the anionic coated electrode increases during the initial 40s and then remains stable, while that for the bare Zn electrode keeps increasing over 180s. According to previous research, the change of current with time at a constant overpotential represents the increase of effective nucleation sites for Zn^2+^.[^6^, ^16^] Therefore, Zn^2+^ could homogeneously nucleate and deposit on the SA‐coated Zn electrode, which is a 2D diffusion. In contrast, there is a 3D diffusion of Zn^2+^ on the bare Zn electrode. The effective nucleation sites on the bare Zn electrode are constantly increasing indicating the aggregation of Zn^2+^ on the electrode, which would lead to the formation of Zn dendrite. To understand the reaction kinetics of Zn^2+^, we computed the interface activation energy (*E*_a_) to examine the impact of the in situ SEI layer on the desolvation process. This calculation was performed using the Arrhenius equation (Figure 1f). Here, *R*_ct_ represents the interfacial resistance, which was tested through electrochemical impedance spectroscopy (Figure S5). The Arrhenius equation takes into account the frequency factor (*A*), gas constant (*R*), and absolute temperature (*T*).[^4b^, ^17^] The in situ SEI layer could reduce the *E*_a_ for Zn^2+^ diffusion from 54.37 kJ mol^−1^ to 12.80 kJ mol^−1^ by 76.5 %, illustrating that it could effectively coordinate the Zn^2+^ solvent sheath and support the uniform Zn^2+^ plating/stripping.
(1)

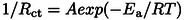




**Figure 2:**
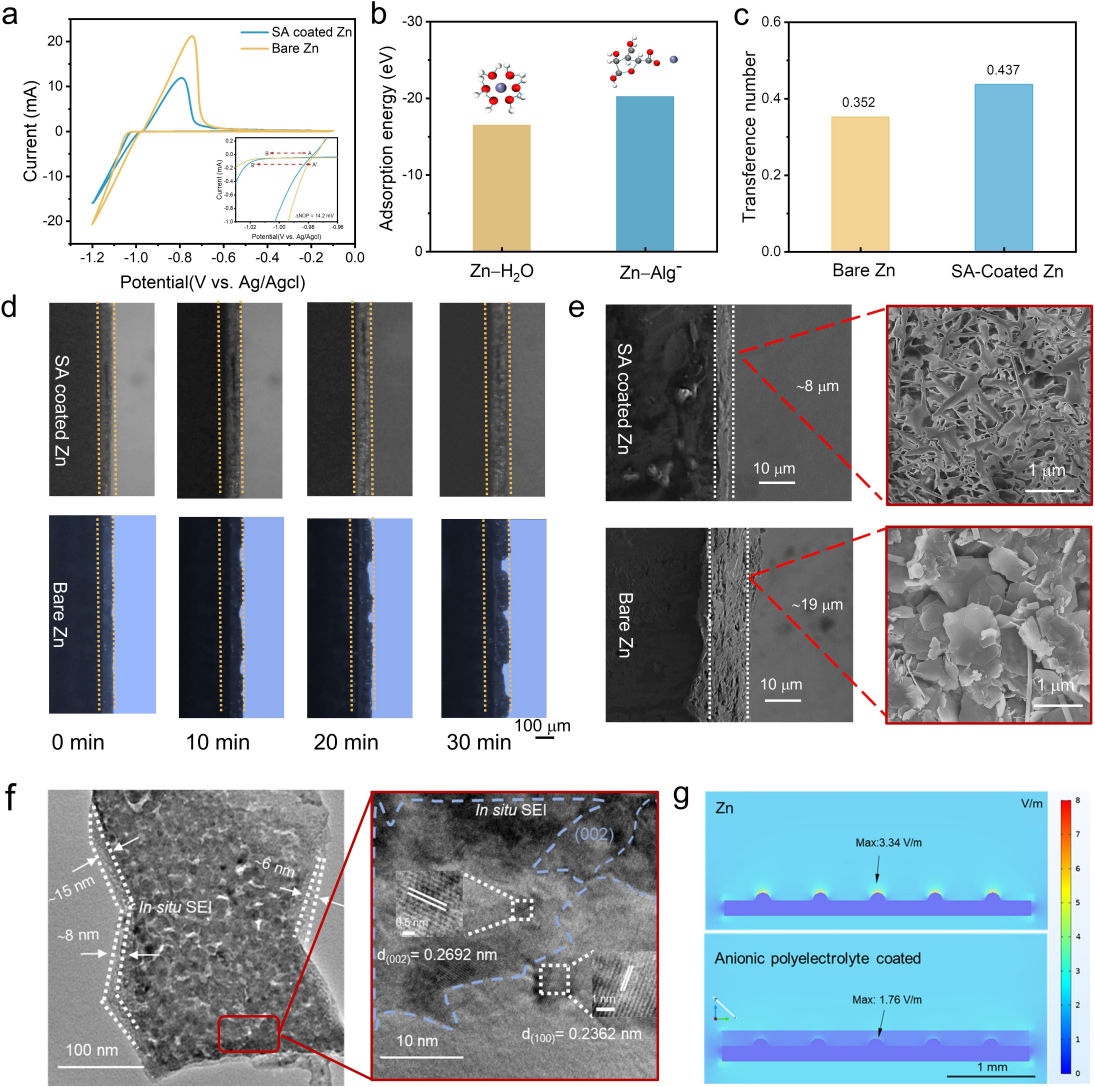
Effect of the in situ SEI layer. (a) Cycling voltammogram (CV) curves of SA‐coated Ti electrode and Bare Ti electrode at a scan rate of 2 mV s^−1^. (b) The adsorption energy for the Zn‐H_2_O and Zn‐Alg^−^ systems with Zn^2+^ solvation structures. (c) Zn^2+^ transference number for bare Zn and SA‐coated Zn anode. (d) In situ optical microscope images of Zn^2+^ plating on the SA‐coated Zn electrode and the bare Zn electrode at a current density of 10 mA cm^−2^. (e) Cross‐sectional and horizontal view of SEM images for bare and SA‐coated Zn anode after 50 cycles (at 0.5 mA cm^−2^ and 0.5 mAh cm^−2^). (f) TEM images for SA‐coated Zn anode after 50 cycles. Enlarged area clearly shows the Zn(002) region. (g) Simulated electric field distribution for bare Zn and SA‐coated respectively with uniform distributed Zn seeds.

To visualise the surface plating mechanism, the Zn^2+^ plating process on both electrodes was recorded through the optical microscope (Figure 2d). During 30‐min plating, SA‐coated Zn exhibited smooth plating, while for the bare Zn, a fluffy plating was obtained. Regarding the stripping mechanism, a significant hydrogen evolution reaction (HER) is observed on the bare Zn electrode within 30 minutes as shown in Figure S6; in contrast, the surface of the SA‐coated Zn electrode keeps smooth and homogeneous morphology without obvious HER, which further illustrates that the in situ SEI layer could improve the thermodynamic stability of Zn^2+^ and mitigate the corrosion and passivation issues of the anode. SEM images (Figure 2e) for the anionic polyelectrolyte‐coated Zn electrode further consolidate the observation SA‐coated Zn anode exhibits uniform and dendrite‐free morphology compared to the bumpy surface of the bare Zn anode. Furthermore, the electric field simulated for the anionic‐coated Zn anode (Figure 2g) indicates a uniform electric field, unlike the “tip effect” observed in the case of the bare Zn anode resulting in a high areal current density for discharging. The presence of the hydrogel layer shields the Zn seeds and results in an even plating and less corrosion.

The cycled bare Zn electrode shows a significantly uneven surface with obvious dendrite formation. In contrast, the cycled SA‐coated electrode exhibits a smooth cross‐linked structure without the dendrite formation. The in situ SEI layer with anionic polyelectrolyte results in an accelerated channel that would allow Zn^2+^ deposition on the interface to form a uniform and dense Zn flake layer. Regarding the side‐view, the thickness of the deposited Zn on the SA‐coated electrode is about 11 μm thinner than that on the bare Zn electrode after 50 cycles of galvanostatic charging/discharging, which indicates that the in situ formed SEI layer could lead to a denser deposition of Zn^2+^ on the anode. The in situ formed interface also regulates the diffusion and the nucleation process of Zn^2+^ to induce a 2D uniform plating/stripping (Figure 1e). TEM images (Figure 2f and Figure S15b) for bio‐inspired anionic layer coated Zn after cycling further prove the SEI layer formation, from which a ≈15 nm thickness layer is detected at the interface. As for the enlarged area, the (002) crystal plane dominated Zn was plated beneath the SEI layer, which accounts for 50.26 % of the areal ratio (Figure 2f and Figure S14).

To exploit the nucleation process, The Zn^2+^ deposition behaviours on the Ti foil were also observed (Figure 2a). The nucleation overpotential (η ) after SA coating is decreased from 42.2 mV to 28.0 mV by 14.2 mV, suggesting that the in situ SEI layer could reduce the nucleation energy. It is regarded as the extra potential required to initiate the formation of bubbles or solid phases at the surface of an electrode during an electrochemical reaction, which is the excess energy required to form the first nucleus of a new phase on an electrode surface.^[18]^ Attribute to the classic nucleation theory shown below, where $\gamma_{s}$ is the surface energy, $k_{B}$ is the Boltzmann's constant and *T* is the temperature, heterogeneous nucleation free energy, Δ$G_{Het}$ , is inversely proportional to nucleation overpotential and nucleation rate, $J_{0}$ , is proportional to the exponential of the negative activation free energy for nucleation, hence, a reduced nucleation free energy could improve the efficiency of nucleation. As clarified by Zhang, a greater *J* would initiate abundant nuclei with a smaller size.^[19]^ Referring to the calculated surface energy before and the measured nucleation overpotential, the ratio of nucleation‐free energy for SA‐treated Zn to the bare Zn is 1 : 34, which indicates that the in situ SEI layer exhibits lower nucleation energy resulting in an increased nucleation rate. The −COO^−^ groups provide an acceleration channel for Zn^2+^ deposition improving the selectivity at the SEI interface. The Zn transference number shown in Figure 2c and Figure S4 further convinces this fact, where SA‐treated Zn exhibits a 24.1 % larger Zn transference number compared to the pristine Zn anode.^[20]^

(2) $$\Delta G_{Het}\propto \frac{16\pi \gamma_{s}^{3}}{3\eta^{2}}$$

(3) $$J_{0}\propto \exp\;\left(\frac{-\Delta G_{Het}}{k_{B}T}\right)$$

Density functional theory (DFT) was performed to analyze the effect of the in situ SEI layer on the Zn^2+^ transport. The adsorption energy of anionic zinc alginate (Zn‐Alg^−^) is lower than that of Zn‐H_2_O, which are −20.22 eV and −16.50 eV, respectively (Figure 2b). Compared with the water molecule, Zn^2+^ prefers to coordinate with the anionic alginate, so the SEI layer could decrease the diffusion barrier and facilitate the desolvation process of Zn^2+^.[^7^, ^21^] As shown in Figure 2b, the Zn^2+^ solvation structure in the Zn‐H_2_O system is [Zn(H_2_O)_6_]^2+^, while in the Zn‐Alg^−^ system, according to the DFT simulation, the Zn^2+^ solvent sheath would be regulated by the SEI layer to form a [Zn(H_2_O)_4_⋅Alg]^+^ solvation structure.

**Figure 3:**
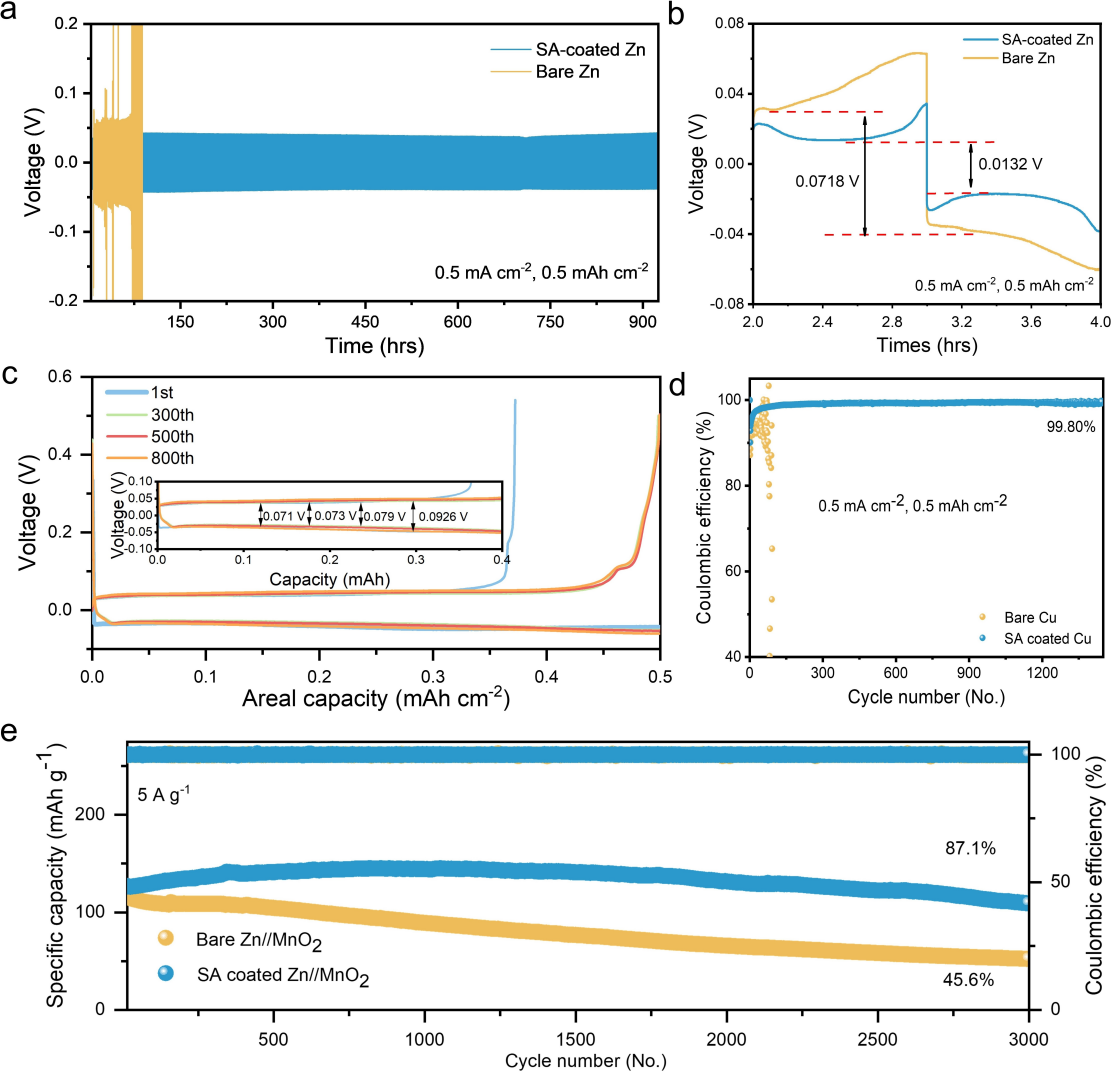
Enhancement of the electrochemical performance using the SA‐coated Zn. (a) The galvanostatic cycling performance of the SA‐coated and bare Zn symmetric cells (current density: 0.5 mA cm^−2^ and capacity density: 0.5 mAh cm^−2^). (b) The corresponding voltage profile for SA‐coated Zn and bare Zn. (c) The corresponding CE voltage profile at various cycles. (d) The CE performance of the Zn^2+^ plating/stripping on the SA‐coated and bare Cu electrode (current density: 0.5 mA cm^−2^ and capacity density: 0.5 mAh cm^−2^. (e) The cycling performance of the battery assembling with the SA‐coated Zn electrode and bare Zn electrode at the current density of 5 A g^−1^.

The stability of the Zn anode was evaluated by long‐term galvanostatic cycling of a symmetric Zn cell (Figure 3a). The in situ SEI layer could effectively improve the electrochemical stability of the Zn^2+^ plating/stripping. The symmetric cell assembled with the SA‐coated electrode exhibits excellent stability around 1,000 h at 0.5 mA cm^−2^ and 0.5 mA cm^−2^, while the cell assembled with the bare Zn would suffer the short circuit start at 30 h with a sudden voltage spike and eventually culminating at 80 h (Figure 3a). The voltage difference of the SA‐coated electrode is 0.0132 V, which is lower than that of the bare Zn electrode (0.0715 V), indicating the good stability of the interface during plating/stripping (Figure 3b). To unveil the stringent conditions, higher depth of discharge (DOD >50 %) and current densities were also examined. As shown in Figure S10b, the SA‐coated Zn anode exhibits a stable Zn stripping/plating behavior with a potential difference of around 0.114 V under 10 mA cm^−2^ and 10 mAh cm^−2^ and high DOD:57.1 %. Even under a high current density of 20 mA cm^−2^, SA‐coated Zn still has a stable performance over 250 hours with a constant potential difference around 0.27 V (Figure S10d). The symmetric cell assembled with the SA‐coated electrode also could keep excellent stability over 250 h at 20 mA cm^−2^ and 1 mA cm^−2^. Even for the rate performance (Figure S11), SA‐coated Zn exhibits lower potential differences of 0.0861 V, 0.096 V, 0.153 V and 0.195 V respectively from 1 mA cm^−2^ to 10 mA cm^−2^ compared to 0.110 V, 0.143 V, 0.202 V and 0.264 V for that of bare Zn. As shown in Figure 3d, the Coulombic efficiency (CE) was analysed using the Zn||Cu cell. The first cycle CE of the bare Zn electrode is around 88.59 % and drops rapidly due to the short circuit of ≈85 cycles. In contrast, the first cycle CE of the SA‐coated Zn electrode is 90.1 %, and the average CE is up to 99.8 % over 1,400 cycles. The voltage profiles at various cycles show that the overpotential could keep at a low and stable value during the cycling (Figure 3c). The CE performance indicates that the in situ SEI layer could effectively reduce the side reactions. As for the Zn||MnO_2_ battery performance test, the cyclic voltammetry (CV) curve shows that both batteries exhibit similar redox peaks attributed to the intercalation/deintercalation of Zn^2+^/H^+^ (Figure S12). Because the in situ SEI layer could enhance the CE of the anode, the battery assembled with the SA‐coated Zn electrode shows a higher specific capacity at a scan rate from 0.1 A g^−1^ to 5 A g^−1^ (Figure S13). Compared with the battery assembled with the bare Zn anode, the SA battery exhibits an excellent specific capacity of 109.3 mAh g^−1^ with 87.1 % capacity retention over 3000 cycles at the current density of 5 A g^−1^ (Figure 3e), which indicates that the effect of the in situ SEI layer on regulating the diffusion of Zn^2+^ could enhance the stability and capacity of the battery. To examine the practical conditions, high mass loading cathode and low N/P ((N/P ratio, the ratio of negative electrode capacity to positive electrode capacity) ZIBs were performed as shown in Figure S17. As analysed by Pan,^[27]^ N/P ratio in the range of 2–5 is practical for ZIBs, N/P around 3 is selected in the test. With an increased mass loading, Zn||MnO_2_ exhibits a less cycling stability, which is a common phenomenon as reported. While for ZIB with SA‐coated Zn anode, it still exhibited a 91 % CE over 60 cycles with loading mass 15 mg cm^−2^ compared to 38 % CE for bare Zn. For mass loading with a lower N/P ratio (≈3), Zn anode suffers from limit conditions in plating/stripping and dendrite formation. As shown in Figure S17a, SA‐coated Zn protect the Zn anode from quick decay. We have also examined the performance using VO_2_ cathode. As shown in Figure S17d, SA‐coated Zn exhibits a CE with 96.9 % compared to bare Zn with 59.1 %. Moreover, we also examined the battery performance in soft‐pack pouch cells. As displayed in Figure S17b and S17e, the SA‐coated Zn anode further facilitates stable pouch cell performances over 40 cycles.

**Figure 4:**
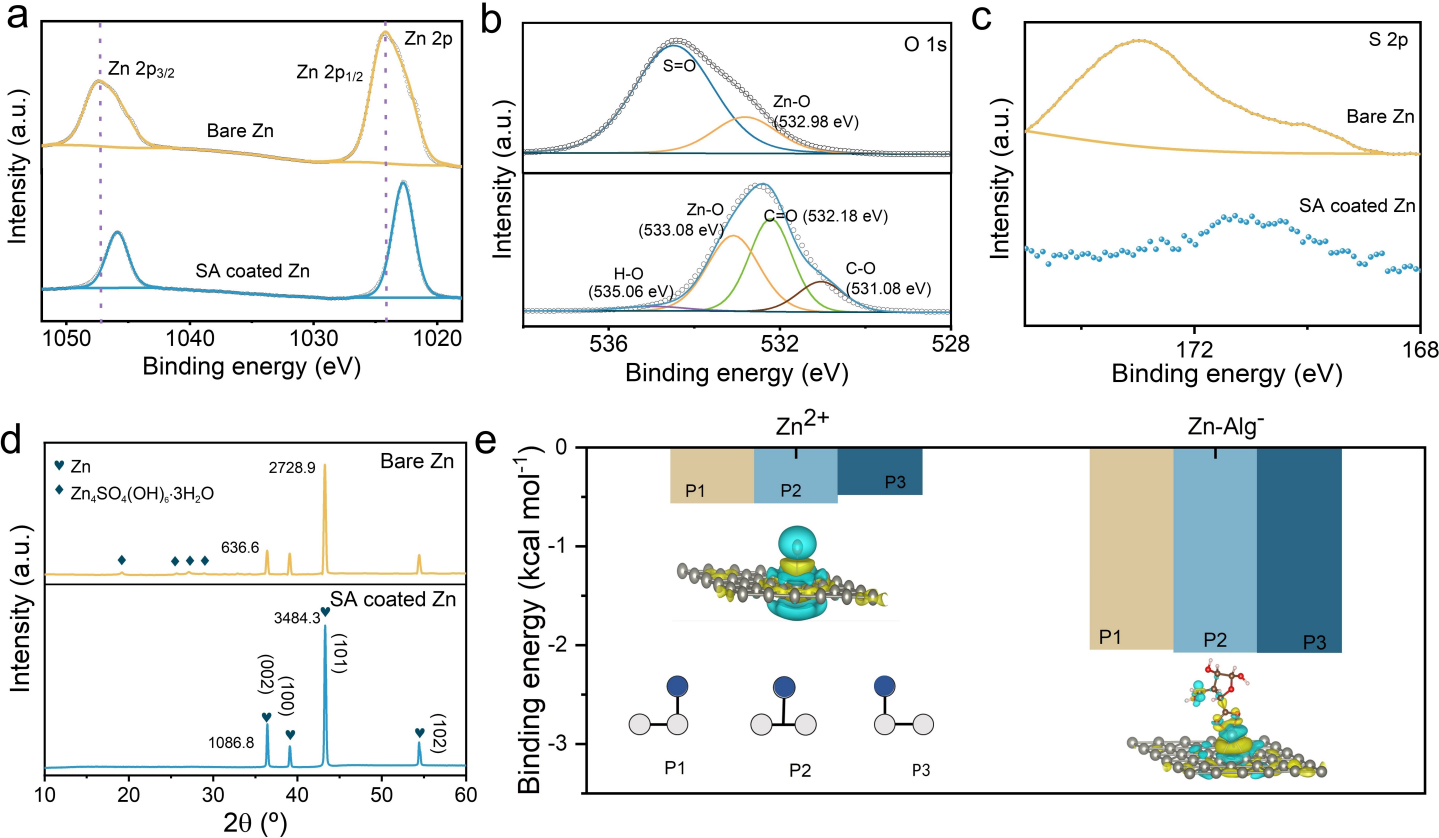
Characterization of the in situ SEI layer to inhibit side reactions. XPS spectra of Zn 2p (a), O 1s (b), and S 2p (c) for bare Zn electrode and SA‐coated Zn electrode after 50 cycles (current density: 0.5 mA cm^−2^ and capacity density: 0.5 mAh cm^−2^). (d) XRD pattern of the SA‐coated electrode and the bare Zn electrode after 50 cycles (current density: 0.5 mA cm^−2^ and capacity density: 0.5 mAh cm^−2^). (e) The binding energy of Zn^2+^ and Zn‐Alg^−^ at the top site (P1), the bridge site (P2) and the top side (P3) of the Zn (002) lattice plane. The grey circles represent the Zn anode structure of Zn (002), and the blue circles represent the potential plating ionic groups of Zn^2+^ and Zn‐Alg^−^.

A series of characterizations were used to analyze the mechanism of the in situ formed SEI layer to inhibit the dendrite growth and the by‐product formation. To analyze the side reactions on the anode, the crystallinity and chemical composition of the Zn electrode after cycling was investigated by X‐ray photoelectron spectroscopy (XPS) and X‐ray diffraction (XRD). As shown in Figure 4a, the peaks of Zn 2p are assigned to Zn 2p_3/2_ and Zn 2p_1/2_, respectively. Compared with the bare Zn electrode, the binding energies of Zn 2p_3/2_ and Zn 2p_1/2_ for the SA‐coated Zn electrode are reduced to a relatively lower level, due to the in situ SEI layer regulating the reaction process of Zn^2+^ at the anode interface.^[22]^ According to the O 1s (Figure 4b) and S 2p spectra (Figure 4c), the peaks of O 1s at 532 eV and 534 eV are attributed to the Zn−O and S−O bonds, respectively.^[23]^ S 2p has not been detected on the SA‐coated electrode after cycling, whereas the S−O bond at 172 eV was detected on the bare Zn anode. The XRD patterns further confirm the composition of the Zn anode after cycling (Figure 4d and Figure S7). The SA characterization peaks are around 15.2° and 27.4°, and the diffraction peak of the by‐product (Zn_4_SO_4_(OH)_6_⋅3H_2_O, ZHS) is not detected on the SA‐coated electrode after the constant cycling. The results from FIB‐SEM EDS mapping shown in Figure S9 further revealed that there is less ZHS in the cross‐sectional SEI layer compared to the bare Zn anode after cycling. In addition, the intensity of the Zn (002) crystal plane of the SA‐coated Zn electrode is stronger than that of the bare Zn electrode after 50 cycles. To quantify the intensity difference, Harris′ method was applied by normalising the intensity of a particular plane to the sum of the rest intensities regarding the standard of JCDPS 04‐0831.^[24]^ As displayed in Table S1, the intensity ratio of (002) for SA‐coated Zn is 0.795 compared to 0.555 for pristine Zn further confirming the domination of the parallel plating. This is consistent with the results from the TEM images after cycling that illustrate before. It could be known that the in situ SEI layer could construct the Zn^2+^ plating/stripping on the (002) plane and effectively inhibit the passivation of the Zn anode. The binding energy for Zn^2+^ and Zn‐Alg^−^ at the top and bridge sites (shown in the inner Figure 4e) of the Zn (002) crystal plane was also calculated by DFT (Figure 4e). Zn‐Alg^−^ exhibits a lower binding energy than Zn^2+^ adsorbing at the bridge site of the (002) plane as the binding energies could reach the lowest, which are −0.563 eV and −2.074 eV, respectively. Therefore, as measured, (002) is dominated in the Zn anode with the SA treatment. The adsorption distance of Zn‐Alg^−^ at the bridge site is 0.110 Å, while the adsorption distance of Zn^2+^ is 0.116 Å (Figure S8). This further confirms that the polyanionic SEI layer facilities Zn diffusion along the interface with a lower interface energy barrier. In consistent with above results, in situ formed layers could more effectively regulate the 2D diffusion of Zn^2+^.

**Figure 5:**
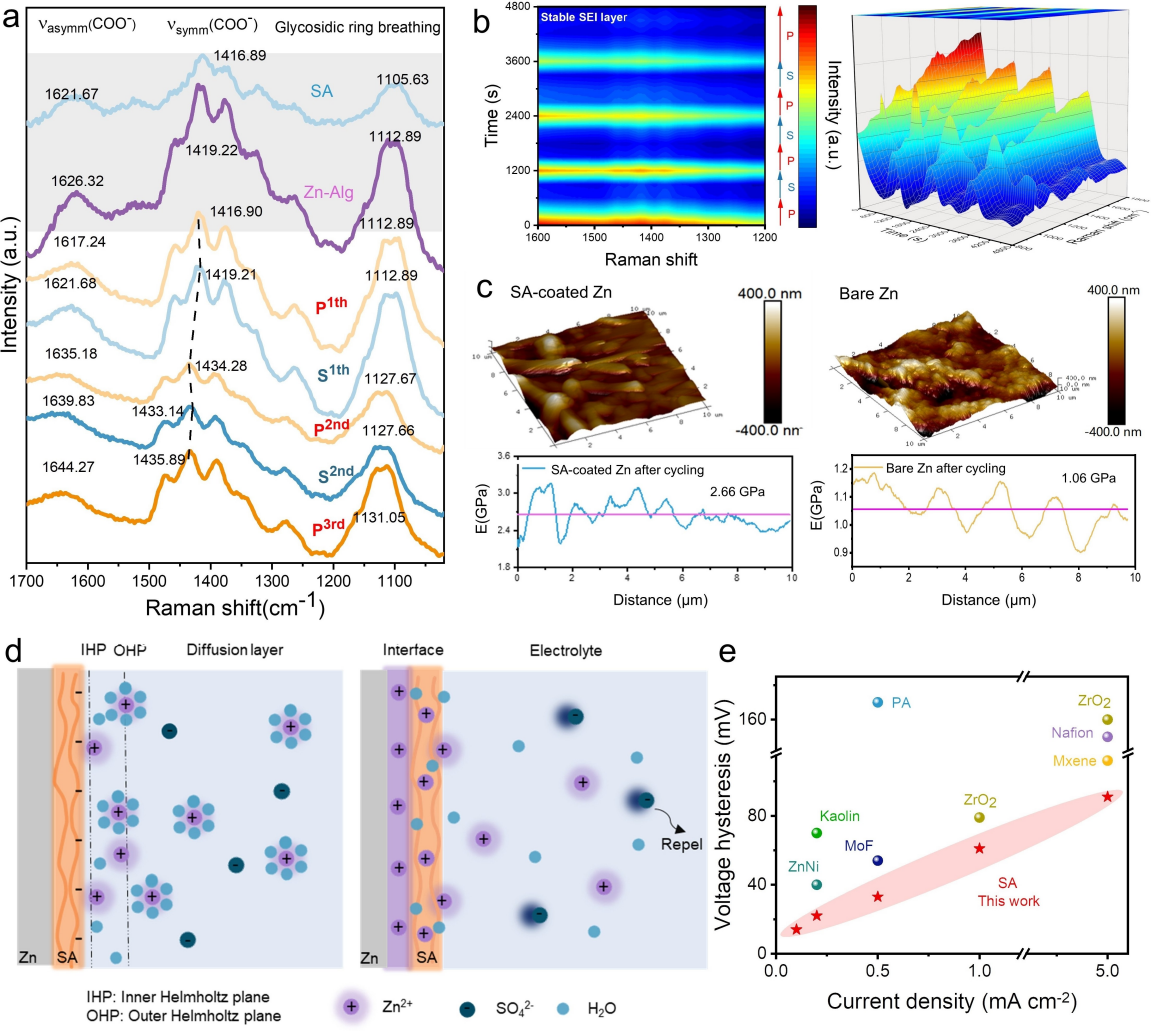
The mechanism of the in situ SEI layer regulating the uniform plating/stripping of Zn^2+^. (a) The in situ Raman spectra of the SA‐coated Zn electrode during the cycling (current density: 0.1 mA cm^−2^ and capacity density: 0.017 mAh cm^−2^). (b) The 2D and 3D images of Raman shift with the cycling time of the SA‐coated Zn electrode. (c) The AFM images and young's modulus measurements for the SA‐coated Zn and bare Zn electrodes before/after 15 cycles (current density: 0.5 mA cm^−2^ and capacity density: 0.5 mAh cm^−2^). (d) The schematic diagram for the process of Zn^2+^ deposition on the bare Zn electrode and the SA‐coated Zn electrode. (e) Comparison of recent anode development and performance regarding voltage hysteresis and current densities.

To verify the stability of the in situ formed SEI layer, in situ Raman spectra were recorded every 300 s during cycling (Figure 5a). After immersing the SA‐coated Zn electrode in 3 M ZnSO_4_ electrolyte for one hour, the band of symmetric COO^−^ vibration shifted from 1416.89 cm^−1^ to 1450 cm^−1^, and the asymmetric COO^−^ vibration band moved from 1621.67 cm^−1^ to 1626.32 cm^−1^, owing to the formation of zinc alginate gelation.^[25]^ Besides, the glycosidic ring breathing mode would also shift to a higher wavenumber (from 1105.63 cm^−1^ to 1112.89 cm^−1^), combined with the FTIR spectra, which could be attributed to the deformation of the C−C‐H/C−O−H bond during the in situ polymerization.^[26]^ As shown in Figure 5b, a periodic intensity variation in each plating/stripping cycle could be observed. During plating, attributed to the coordination of Zn^2+^ to the COO^−^ groups, the symmetric stable egg‐box structure reduces the polarization, hence a reduced intensity of characteristic bands from 1300 cm^−1^ to 1500 cm^−1^ was attained. While during stripping, the escape of Zn^2+^ further increases the asymmetricity, hence resulting A greater intensity. In the meantime, the band shift could also be observed as shown in Figure 5a. During the plating, the band shift would move to a lower wavenumber, since the Zn^2+^ in the Zn‐Alg^−^ would be reduced to Zn in the interface. Conversely, the band would shift to a higher wavenumber in the stripping process. The highly reversible intensity and shift variation indicates the outstanding stability of the SEI layer. Mechanical properties even convince this fact. As measured by atomic force microscopy (AFM), the mechanical strength of the SEI layer would increase with cycling to some extent. Compared to the bare Zn as shown in Figure 5c, the bare Zn anode surface morphology after cycling is rougher and bumpier compared to the SA‐coated Zn. Moreover, regarding the Young's modulus, SA‐coated Zn exhibits a twice higher magnitude than that of bare Zn after cycling, which indicates the robustness of the hydrogel protective SEI layer of the SA‐coated Zn. Based on the characterization and simulation results, the strengthened elasticity of the SA coating layer inhibits the passivation and corrosion of the electrode. As for the bare Zn electrode, Zn^2+^ would aggregate and nucleate on the defect to minimize the surface energy due to the tip effect, which would cause uniform deposition of Zn^2+^ and the dendrite formation. Bio‐inspired anionic coating could effectively modify the defect of the Zn anode, suppressing the tip effect. As stated by Liu et al.,[^30^, ^31^] the presence of polar groups exhibiting varying steric hindrances plays a pivotal role in governing the formation of the SEI. Therefore, for the anionic coating, attraction of −COO^−^ to Zn^2+^ form an in situ SEI layer, allowing the formation of the acceleration channel for plating. The negatively charged interface reduces the interface activation energy and repels SO_4_^2−^ in the electrolyte alleviating the formation of ZHS. Thereby, Zn^2+^ ions are well distributed at the inner Helmholtz plane resulting in a uniform denser plating and reducing the tendency of corrosion for the Zn anode (Figure 5d). Compared to other strategies, this work by utilizing bio‐inspired polyanionic electrolyte could offer a merit voltage hysteresis (Figure 5e and Table S2), for which under the same current density, the biomass polyanionic coating exhibits the lowest overpotential. This anionic polyelectrolyte can be applied on both cathode and anode as a universal candidate for interface regulations.

## Conclusion

In summary, the bio‐inspired in situ SEI layer was constructed which is highly reversible and stable. Inspired by seaweeds, anionic polyelectrolyte was coated on the Zn anode inhibiting the passivation and corrosion of the Zn anode, which could in situ polymerize with the Zn^2+^ in the electrolyte to in situ form the SEI layer with excellent mechanical strength. Attribute to the anionic groups of −COO^−^, this SEI layer generates an accelerating channel for Zn deposition with reduced nucleation‐free energy which is only 2.9 % of the energy required for pristine Zn. With the merits in the accelerating channel, the Zn transference number has increased to 0.437 from 0.352, and even a denser deposition layer with the domination of Zn (002) was attained. The anion‐charged surface also regulates the desolvation structure of Zn^2+^ repelling negative SO_4_^2−^ and alleviating the by‐product formation. The well‐distributed Zn^2+^ cations at the inner Helmholtz plane deliver a uniform and denser plating behaviors and reduce the tendency of anode corrosion. The simulated results from finite element analysis further reveals that the maximum electric field of anionic coated Zn is only 52.7 % compared to the pristine Zn anode.

## Conflict of interest

The authors declare no conflict of interest.

1

## Supporting information

As a service to our authors and readers, this journal provides supporting information supplied by the authors. Such materials are peer reviewed and may be re‐organized for online delivery, but are not copy‐edited or typeset. Technical support issues arising from supporting information (other than missing files) should be addressed to the authors.

Supporting Information

## Data Availability

The data that support the findings of this study are available from the corresponding author upon reasonable request.
