# Supplementary material for: Bio‐Inspired Polyanionic Electrolytes for Highly Stable Zinc‐Ion Batteries
Source: Angew Chem Int Ed Engl. 2023 Sep 1;62(41):e202311268. doi: 10.1002/anie.202311268 (PMC10962557; doi:10.1002/anie.202311268)
Supplement: Supplementary file 1 — Supporting Information [file ANIE-62-0-s001.pdf]

Angewandte Chemie
Eine Zeitschrift der Gesellschaft Deutscher Chemiker

Supporting Information

Bio-Inspired Polyanionic Electrolytes for Highly Stable Zinc-Ion Batteries

*H. Dong, X. Hu, R. Liu, M. Ouyang, H. He, T. Wang, X. Gao, Y. Dai, W. Zhang, Y. Liu, Y. Zhou, D. J. L. Brett, I. P. Parkin, P. R. Shearing, G. He**

Supporting information

Experimental section

Preparation of SA-coated Zn anodes

The SA layer was prepared by a simple spin coating method. Around 1 g SA was added in 15 mL distilled water to form the alginate solution. A fixed volume (100 μL, 150 μL and 200 μL) of the alginate solution was spin coated on the Zn foil (thickness: 30 μm) at 1500 rpm for 10s, and then was dried under a UV light about 5 minutes to form the SA layer. The optimized volume of the SA solution for the spin coating was investigated through the galvanostatic charge-discharge (GCD) test of the symmetric battery (Figure S3). The battery prepared with 150 μL SA solution could stably run around 60 h and has a lower voltage difference (~0.1652 V) with the voltage hysteresis of 0.0812 V. Therefore, 150 μL SA solution was used for the preparation of the SA-coated Zn electrode.

Synthesis of the cathode materials

$Na_{0.65}Mn_2O_4\cdot 1.31H_2O$ was synthesised by a facial co-precipitation method. All chemicals were from Sigma-Aldrich. The solution I was prepared by dissolving 15 mmol manganese (II) nitrate tetrahydrate and 55 mmol NaOH into 50 mL deionized water. The solution II was prepared by mixing 12 mL H_2O_2 (30 wt%) with 90 mL deionized water. Both solutions were stirred under ambient environment for 10 minutes. After that, the solution II was quickly added to solution I with vigorous magnetic stirring for 10 min, which was moved in an ice bath for 24 h afterwards. The final product was washed by distilled water for 5 times and freeze-dried for 24 h.

Battery Assembly

As for the full battery assembly, the as-prepared $Na_{0.65}Mn_2O_4\cdot 1.31H_2O$ was mixed with carbon black and PVDF in NMP solution at a weight ratio of 7:2:1. After grounding, the mixture was coated evenly on the hydrophilic carbon fiber paper (loading mass: 1.5-2 mg cm^{-2}) and dried in a vacuum oven at 65 °C overnight. After that, the dried cathode was assembled in a CR2032-coin cell with the SA-coated Zn anode, used 3 M $ZnSO_4$ and 0.2 M $MnSO_4$ as the electrolyte. As for the symmetric battery assembly, the SA-coated Zn foil was assembled in the coin cell as both cathode and anode with 3 M $ZnSO_4$ as the electrolyte.

Materials Characterisation

X-ray diffraction (XRD) patterns were obtained by a STOE SEIFERT diffractometer under the radiation source of Cu metal. Scanning electron microscopy (SEM) images were obtained by a JEOL JSM-6701F Field Emission Scanning Electron Microscope (JEOL, Japan) operated at the acceleration voltage of 10 kV. The SEM coupled Energy-dispersive X-ray spectroscopy (EDX) images were obtained by a Carl Zeiss EVO MA10 (Carl Zeiss AG, Germany) and UItim Extreme Silicon Drift Detectors (Oxford Instrumental plc, UK). X-ray photoelectron spectroscopy (XPS) was operated on a monochromatic Al-Kα source corrected with Thermo-Scientific Kα spectrometer (Thermo Fisher Scientific, U.S.). To calibrate the photoelectron energy, C 1s peak at 284.8 eV was used as a standard reference, and the XPS data was analyzed by the CasaXPS software. Fourier transform infrared spectroscopy (FTIR) was measured by Shimadzu IRTracer-100 with the wavenumber from 400 to 4000 cm^{-1}. The Raman data was obtained by a Thermo Scientific™ DXR3 Raman Microscope with a laser wavelength of 532 nm.

Electrochemical Characterisation

The battery galvanostatic charge-discharge test was measured by NEWARE battery testing systems. The cyclic voltammetry (CV) and electrochemical impedance spectroscopy (EIS) were investigated by the VMP3 Biologic potentiostat.

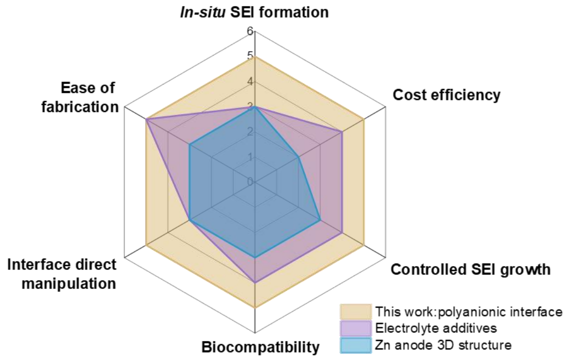

Figure S1. Radar diagram of comparing different strategies to stabilize the Zn anode

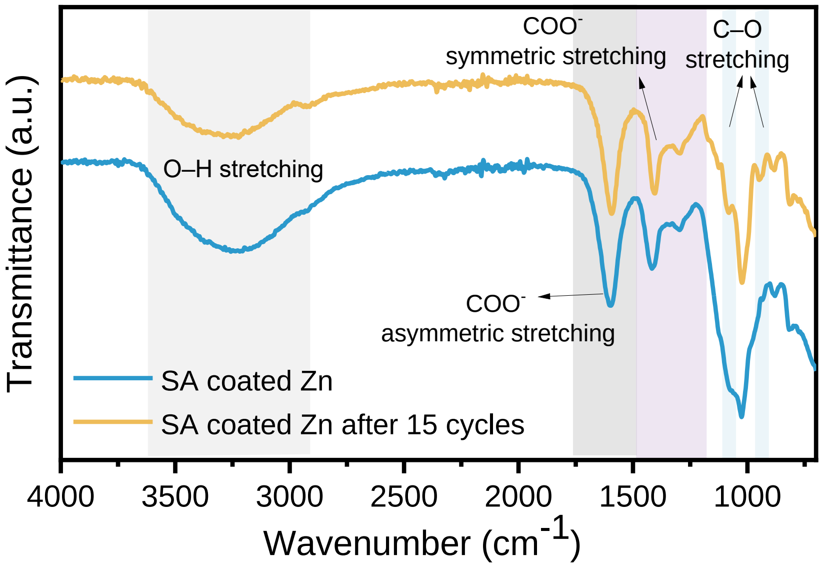

Figure S2. Fourier transform infrared (FTIR) spectra of the SA-coated Zn electrode before and after 15 cycles.

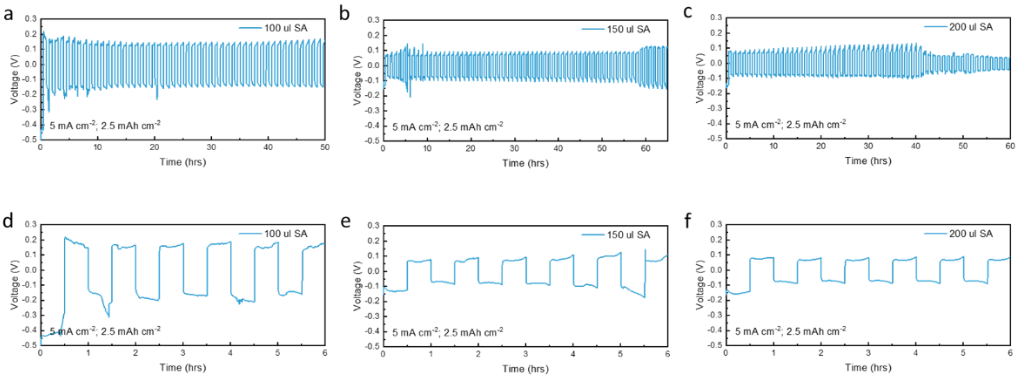

Figure S3. Galvanostatic charge-discharge (GCD) curves of the symmetric battery with different fixed volumes of the SA solution coated Zn foil (current density of 5 mA cm^{-2} and capacity of 2.5 mAh cm^{-2}).

Zinc transference number was also calculated according to the equation as below. The Zn^{2+} transference number (t_+) was measured by a potentiation polarisation (∇V = 5 mV) embedded with electrochemical impedance spectroscopy (EIS) before and after the polarisation. R_o and R_{ss} are resistances for initial and steady-state impedance respectively. I_0 and I_{ss} are initial and steady-state current after 7200 seconds.

$$t_+ = \frac{(\nabla V/I_0 - R_0)}{(\nabla V/I_{ss} - R_{ss})}$$

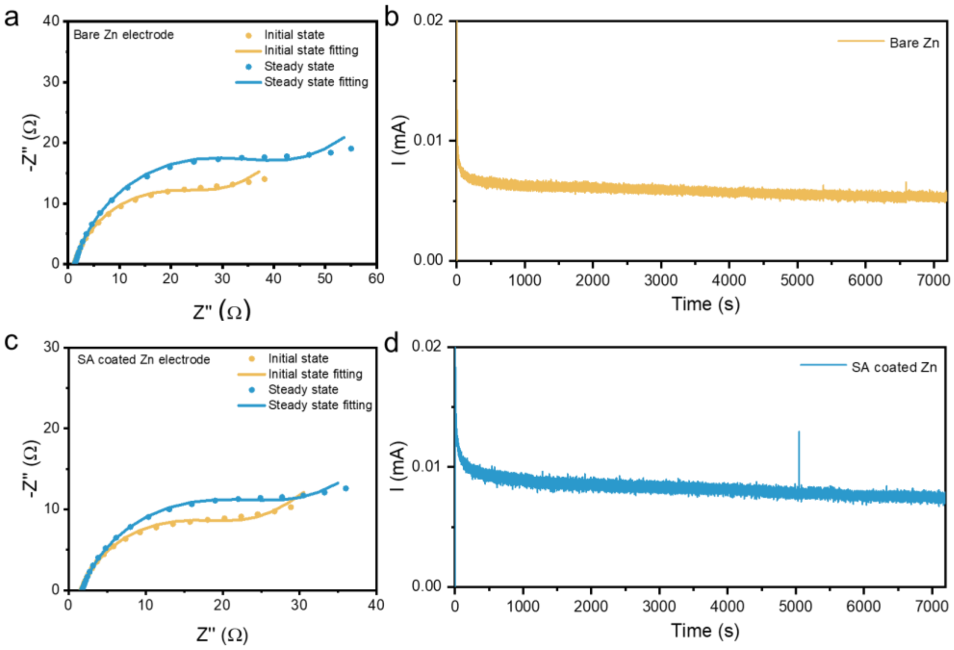

Figure S4. Zn transference number calculation. Bare Zn (a) EIS test before and after potentiostatic polarisation; (b) Current profile of the STE and the aqueous electrolyte in the Zn||Zn symmetric cell under potentiostatic polarisation (∇V= 5 mV) with the reference electrode Ag/AgCl. Anionic coated Zn anode (c) EIS test before and after potentiostatic polarisation; (d) Current profile of the STE and the aqueous electrolyte in the Zn||Zn symmetric cell under potentiostatic polarisation (∇V= 5 mV) with the reference electrode Ag/AgCl

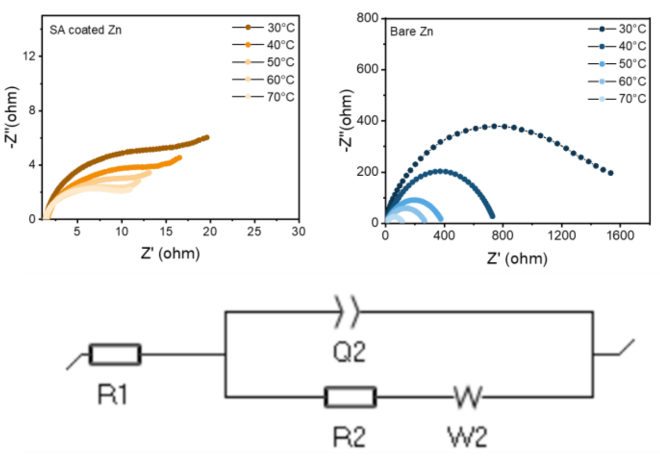

Teperature (℃)	30	40	50	60	70
Bare Zn Rct (ohm)	1599	748.3	391.6	275.4	119.7
SA coated Rct (ohm)	13.97	11.32	9.116	8.573	7.697

Figure S5. EIS test at different temperatures of the SA-coated Zn electrode and Bare Zn electrode.

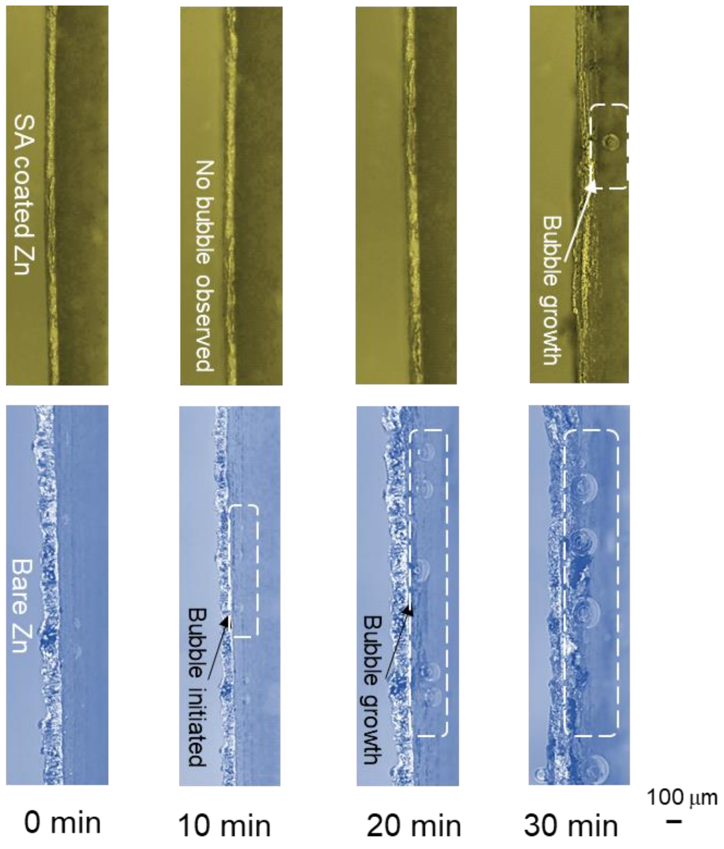

Figure S6 Gas bubble generation for SA-coated Zn and bare Zn respectively.

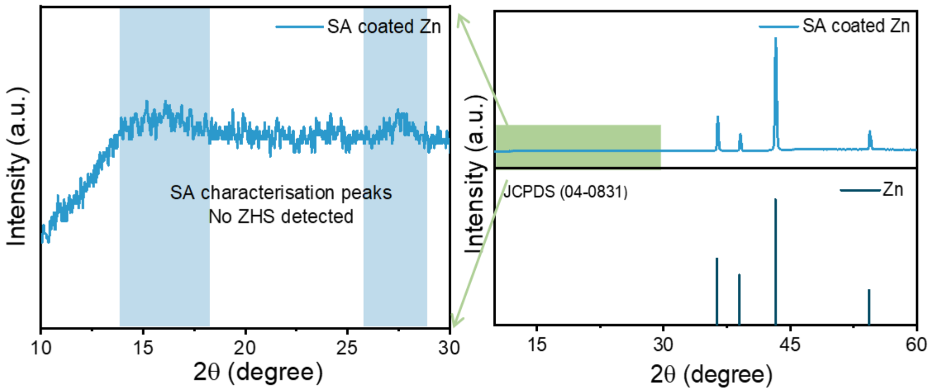

Figure S7. XRD pattern of the SA-coated Zn electrode after 50 cycles.

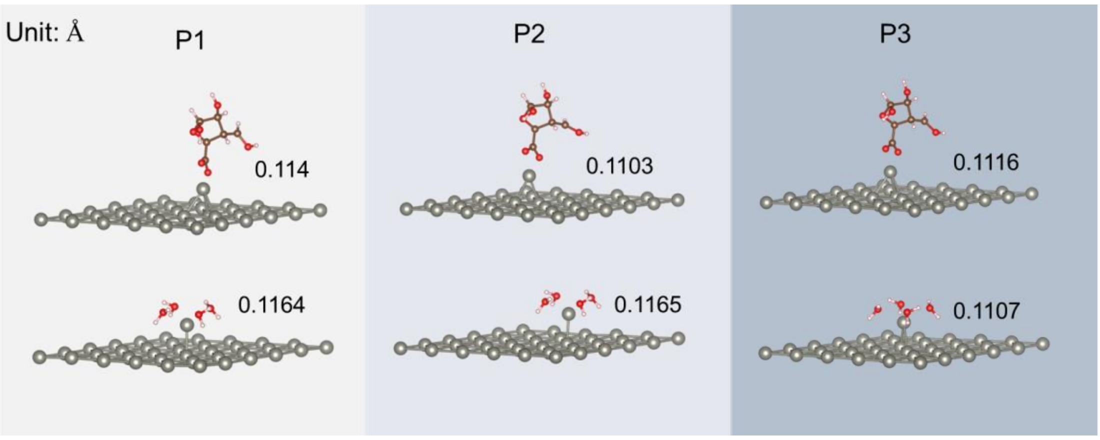

Figure S8. DFT simulation for the adsorption distance of Zn^{2+} and Zn-alg^{-} at the top, bridge, and bottom site on the Zn (002) plane. Grey balls represent the Zn atoms in Zn (002) plane.

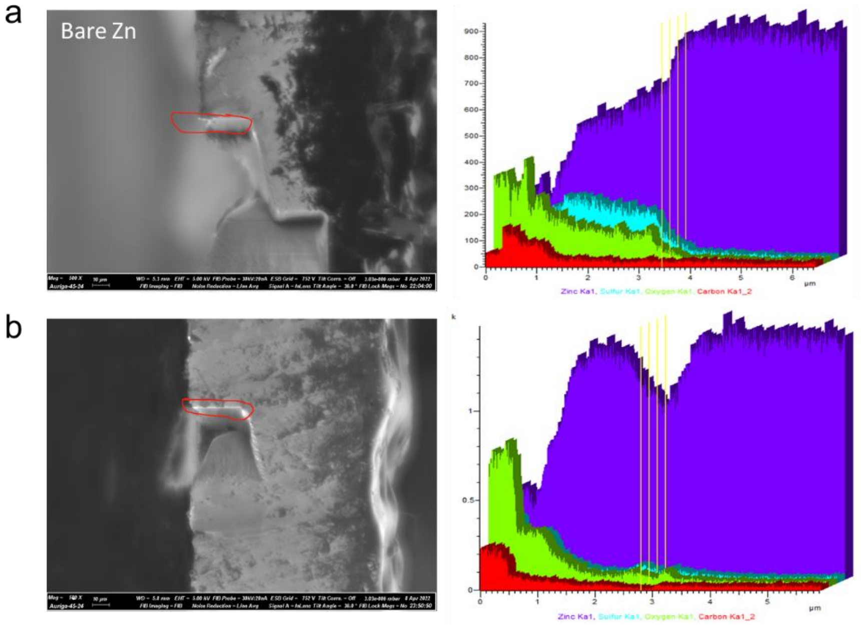

Figure S9. FIB-SEM after cycling (50 cycles 0.5 mA cm^{-2} and 0.5 mAh cm^{-2}). (a) Bare Zn after cycling. (b) SA-coated Zn after cycling.

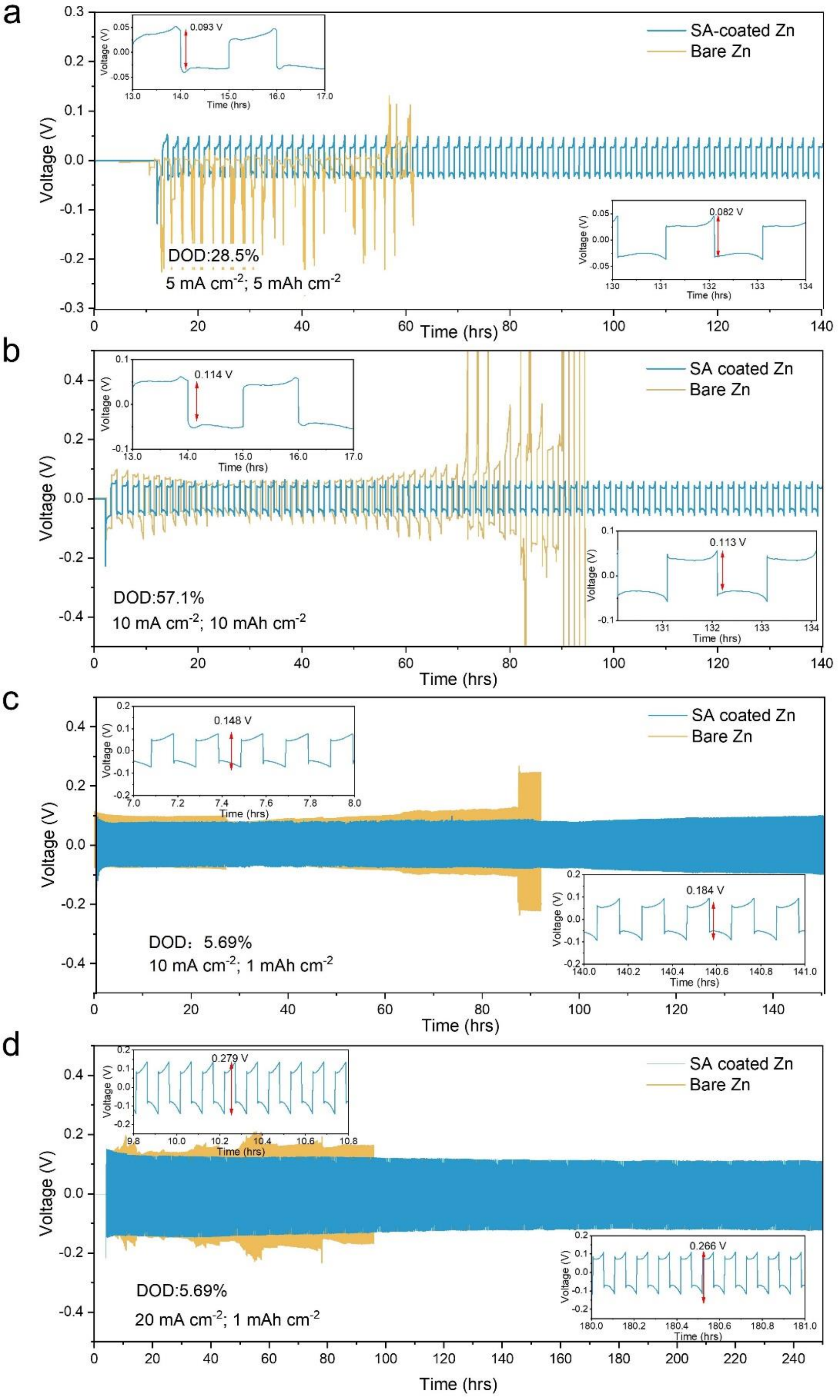

Figure S10. GCD curves of the SA-coated Zn symmetric cell at (a) 5 mA cm^{-2} and 5 mAh cm^{-2} (b) 10 mA cm^{-2} and 10 mAh cm^{-2} (c) 10 mA cm^{-2} and 1 mAh cm^{-2} (d) 20 mA cm^{-2} and 1 mAh cm^{-2}. For these tests, 12 cm diameter Zn foils with 30 μm thickness have been utilised which exhibit a theoretical capacity of 17.5 mAh cm^{-2} (19.8 mAh in total). Depth of discharge (DOD) was calculated based on the theoretical capacity.

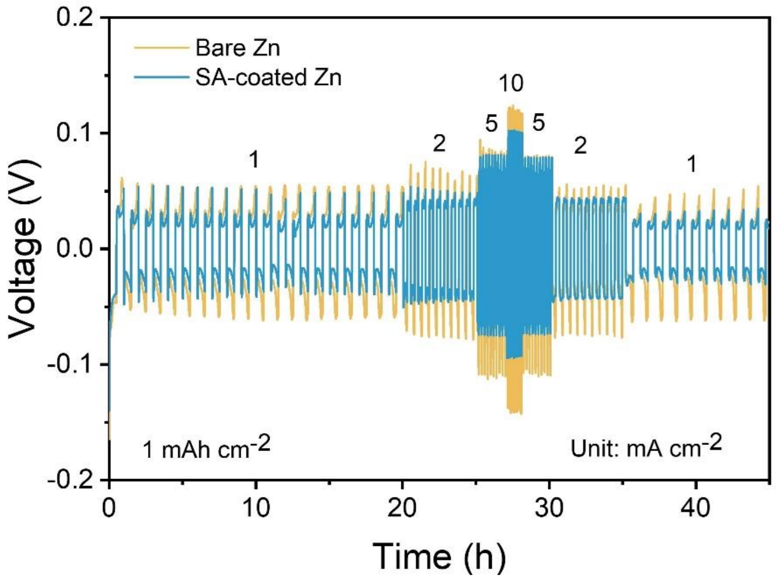

Figure S11. rate performance of the symmetric Zn||Zn cells.

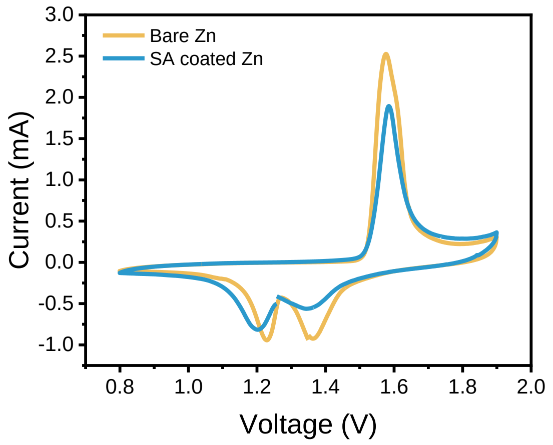

Figure S12. CV curves of the full-cell battery assembled with the SA-coated Zn electrode and Bare Zn electrode at a scan rate of 1mV s^{-1}.

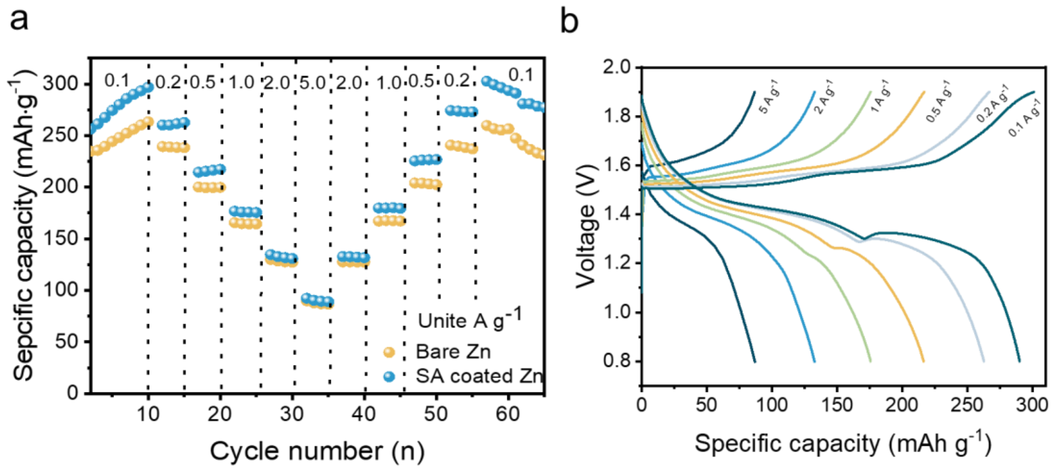

Figure S13. (a) The rate performance of the SA-coated Zn electrode and Bare Zn electrode assembled full-cells at scan rates of 0.1, 0.2, 0.5, 1, 2, 5 A g^{-1}. (b) Voltage-capacity profile at different rates.

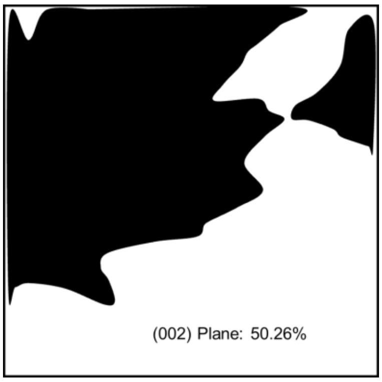

Figure S14. Computational calculation of (002) plane area

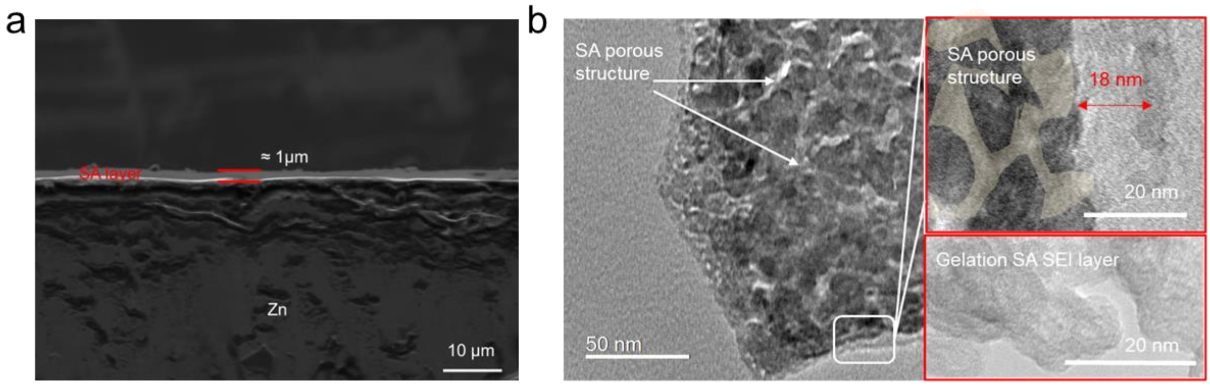

Figure S15. Cross-sectional view for SA-coated Zn. (a) SEM image for SA-coated Zn before cycling; (b) TEM image for SA after cycling.

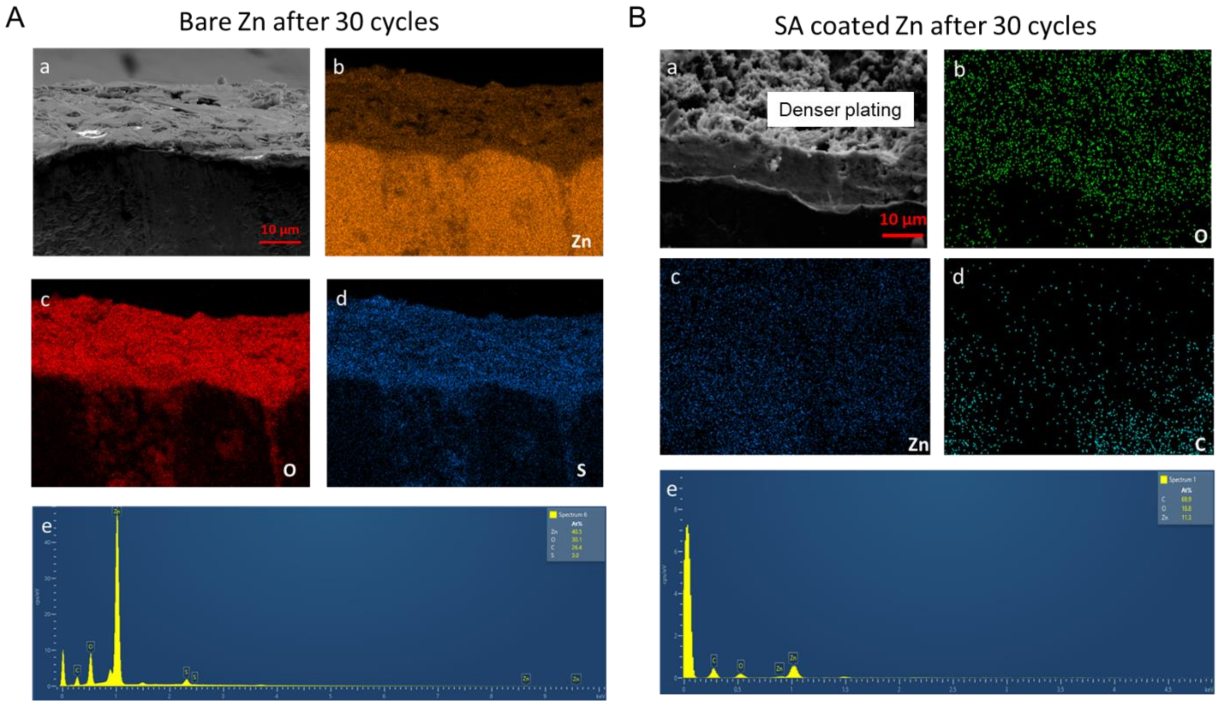

Figure S16. SEM and EDS for bare Zn and SA-coated Zn respectively.

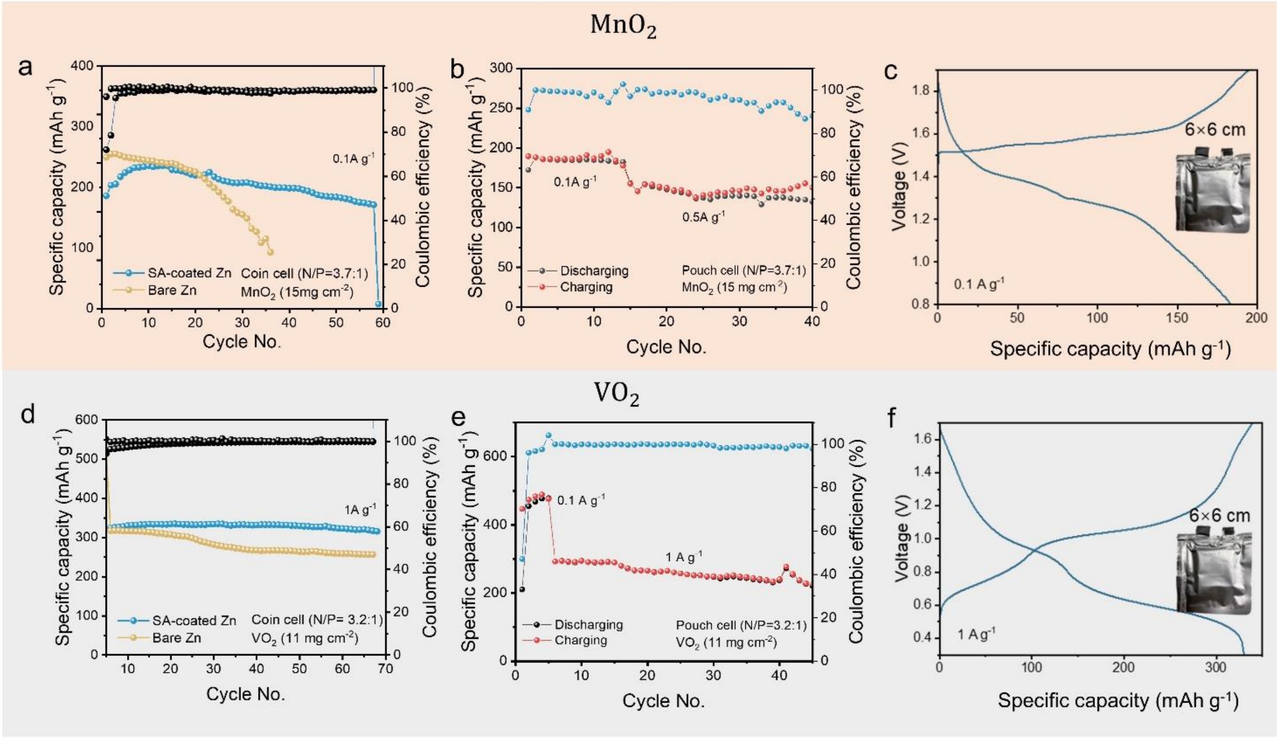

Figure S17. Low N/P ratio and soft-pack pouch cell performance for SA-coated Zn. (a)-(c) Zn||MnO_2 battery performance in coin-cell and pouch cell respectively. (d)-(f) Zn||VO_2 battery performance in coin-cell and pouch cell respectively. Zn foil with 30μm thickness is utilised.

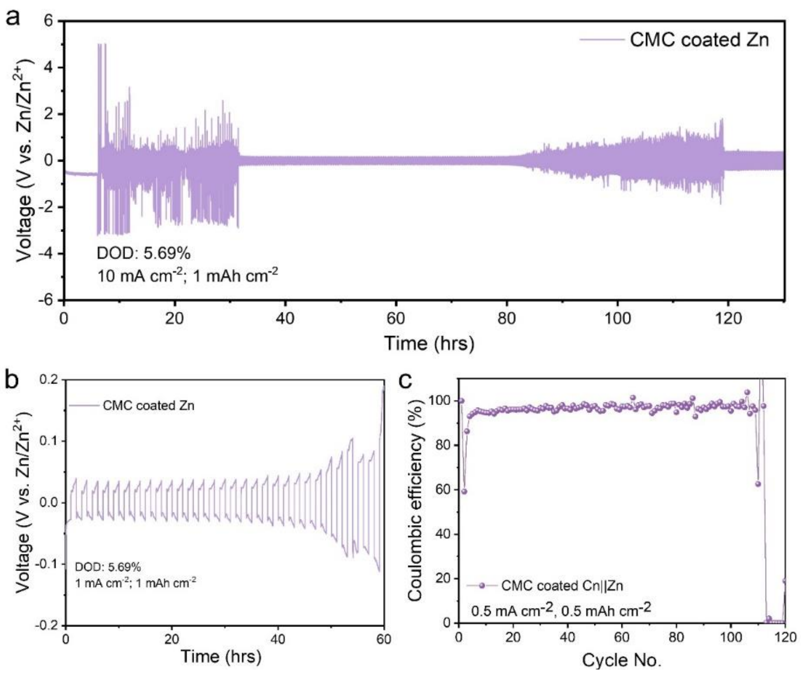

Figure S18 CMC coated Zn electrochemical performance. (a) CMC coated Zn||Zn performance under 10 mA cm^{-2}; 1 mAh cm^{-2}. (b) CMC coated Zn||Zn performance under 1 mA cm^{-2}; 1 mAh cm^{-2}. CMC coated Cu||Zn performance under 0.5 mA cm^{-2}; 0.5 mAh cm^{-2}.

Table S1

**Plane**	**SA-coated Zn**	**Bare Zn**
002	0.795	0.555
100	0.505	0.693
101	0.742	0.730

Harris method:

$$P_{hkl} = \frac{I_{hkl}}{\sum I_{hkl}} \cdot \frac{\sum I'_{hkl}}{I'_{hkl}}$$

I: synthesis intensity

I': standard intensity

Table S2

**Coating layer**	**Current density (mA cm^{-2})**	**Voltage hysteresis (mV)**
SA (This work)	0.1	14
	0.2	22
	0.5	38
	1	61
	5	91
PA[1]	0.5	~170
ZnNi[2]	0.2	~40
kaolin[3]	0.2	~70
MXene[4]	5	112
MoF[5]	0.5	~54
ZrO_2[6]	1	~79
	5	160
Nafion[7]	5	~150

Reference

[1] Z. Zhao, J. Zhao, Z. Hu, J. Li, J. Li, Y. Zhang, C. Wang, G. Cui, *Energy & Environmental Science* **2019**, *12*, 1938-1949.

[2] P. Cao, J. Tang, A. Wei, Q. Bai, Q. Meng, S. Fan, H. Ye, Y. Zhou, X. Zhou, J. Yang, *ACS Applied Materials & Interfaces* **2021**, *13*, 48855-48864.

[3] C. Deng, X. Xie, J. Han, Y. Tang, J. Gao, C. Liu, X. Shi, J. Zhou, S. Liang, *Advanced Functional Materials* **2020**, *30*, 2000599.

[4] N. Zhang, S. Huang, Z. Yuan, J. Zhu, Z. Zhao, Z. Niu, *Angewandte Chemie International Edition* **2021**, *60*, 2861-2865.

[5] C. Wei, L. Tan, Y. Zhang, S. Xiong, J. Feng, *ChemPhysMater* **2022**, *1*, 252-263.

[6] P. Liang, J. Yi, X. Liu, K. Wu, Z. Wang, J. Cui, Y. Liu, Y. Wang, Y. Xia, J. Zhang, *Advanced Functional Materials* **2020**, *30*, 1908528.

[7] Y. Cui, Q. Zhao, X. Wu, X. Chen, J. Yang, Y. Wang, R. Qin, S. Ding, Y. Song, J. Wu, *Angewandte Chemie International Edition* **2020**, *59*, 16594-16601.
